# Characteristics of Out-of-Hospital Cardiac Arrest Trials Registered in ClinicalTrials.gov

**DOI:** 10.3390/jcm13185421

**Published:** 2024-09-12

**Authors:** Jacopo D’Andria Ursoleo, Samuele Bugo, Rosario Losiggio, Alice Bottussi, Viviana Teresa Agosta, Fabrizio Monaco

**Affiliations:** Department of Anesthesia and Intensive Care, IRCCS San Raffaele Scientific Institute, 20132 Milan, Italy; dandria.jacopo@hsr.it (J.D.U.); samuele.bugo@gmail.com (S.B.); r.losiggio@studenti.unisr.it (R.L.); bottussi.alice@hsr.it (A.B.); agosta.viviana@hsr.it (V.T.A.)

**Keywords:** advanced life support, basic life support, cardiopulmonary resuscitation, clinical trials, out-of-hospital cardiac arrest

## Abstract

**Background/Objective:** Out-of-hospital cardiac arrest (OHCA) poses a substantial public health concern. A collective evaluation of clinical trials is crucial for understanding systemic trends and progress within a specific research area of interest, ultimately shaping future directions. We performed a comprehensive analysis of the characteristics of trials in the adult OHCA population registered on ClinicalTrials.gov. **Methods:** Aided by medical subject headings (MeSH), we systematically searched the ClinicalTrials.gov database. Trends over time were assessed with the Cochran–Mantel–Haenszel test. The association between publication year and annual number was assessed with the Pearson correlation coefficient. **Results:** Out of 152 trials spanning the 2003–2023 period, 29.6% were observational and 70.4% were interventional. Compared with the observational trials, interventional trials were more often randomized (RCT) and achieved full publication status in 84% of cases (*p* = 0.03). The primary focus of interventional trials was “procedures” (43%), “devices” (23%), and “drugs” (21%). Observational studies focused on “biomarkers” (16%) and “diagnostic test” (13%) (*p* < 0.001). A decrement in the number of interventional trials with a sample size ≥100 patients across three temporal study points was observed. Nevertheless, published studies predominantly had a sample size ≥100 patients (76%), in contrast to unpublished trials (*p* ≤ 0.001). An increase in the number of interventional studies funded by the “academic/university” sector was also recorded. **Conclusions:** Clinical trials on OHCA primarily involved interventions aimed at treatment and were more often randomized, single-center, with small (<100) sample sizes, and funded by the “academic/university” sector.

## 1. Introduction

Sudden cardiac arrest stands as a primary cause of death, with out-of-hospital cardiac arrest (OHCA) representing a significant contributor to both mortality and morbidity and posing a substantial public health concern [1,2,3]. The incidence of OHCA has consistently increased in recent years, affecting 30 to 97 individuals per 100,000 person-years, despite notable variability in the published literature [4,5]. Moreover, its survival rates remain low, ranging from 4.6% to 16.4% [5].

Over the years, there has been a significant emphasis on improving various aspects related to cardiac arrest and cardiopulmonary resuscitation (CPR). Such an emphasis is mirrored in a significant increase in the number of clinical trials primarily aimed at enhancing OHCA survival rates by validating the effectiveness of different interventions.

While trial characteristics evolve over time [6], and funding and methodological design are determined on an individual trial basis, evaluating trials collectively is crucial for understanding systemic trends and progress within a specific research area of interest.

Despite the pivotal role of OHCA trials in generating practice-defining evidence and their substantial financial and patient costs, to the best of our knowledge, no study to date has either comprehensively characterized OHCA trials or examined how the portfolio of trials has changed over time. Indeed, an in-depth analysis encompassing such an approach has not yet been performed, since previous studies have only focused on providing an overview of a quite limited subset of OHCA trials published in a few indexed and peer-reviewed journals [7,8].

**Figure 1:**
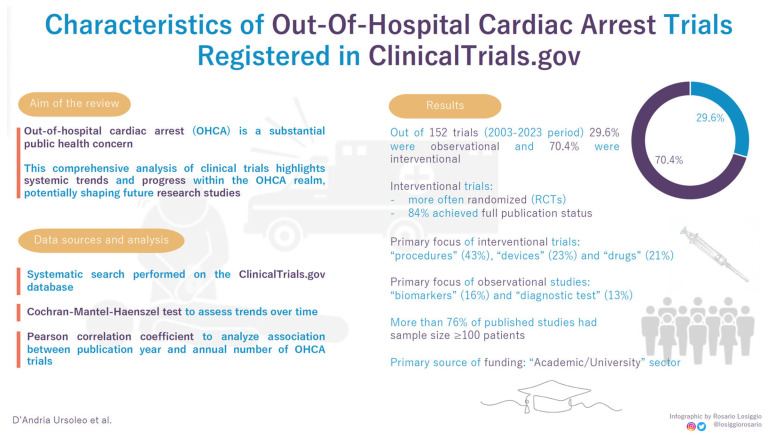
Visual abstract presenting main article structure, objective, research methodology, and results.

Utilizing ClinicalTrials.gov, the most comprehensive publicly accessible registry of interventional clinical trials, we aimed to identify trials focusing on OHCA among the over 480,000 registered clinical research trials. Our analysis considered various characteristics of clinical trial design within OHCA trials, with a specific emphasis on elements crucial for generating reliable evidence (e.g., randomization, multicentric design, enrollment size). Furthermore, we assessed the influence of funding sources on trial methodology and explored the evolving landscape of OHCA trials over time (Figure 1).

## 2. Materials and Methods

### 2.1. Analysis of Data Source, Study Selection and Labeling, Ethical Considerations

On 31 December 2023, the ClinicalTrials.gov database was searched to identify publicly available records of potential OHCA trials. The terms “cardiac arrest” and “out-of-hospital” and their synonyms, “out-of-hospital cardiac arrest”, “out-of-hospital cardiac arrests”, “heart arrest”, and “asystole”, together with the related medical subject heading (MeSH) terms, were reviewed and combined using the Boolean operators “AND” and “OR”.

Because the data were obtained from a public database, the present investigation was exempt from institutional review board approval.

Search results were then downloaded using the Aggregate Analysis of ClinicalTrials.gov (AACT) database tool [9] as a CSV file and then imported into a separate database to facilitate further data filtering, classification, and management.

Two trained and independent investigators (J.D.U. and S.B.) manually reviewed the search results at the official title/study abstract level and, if needed, the detailed description level, to exclude trials that were either ongoing or carried out in the pediatric setting or that lacked information on the type of intervention being tested.

### 2.2. Extraction of Clinical Trials’ Characteristics

The following 11 trial characteristics were extracted from the ClinicalTrials.gov record for analysis: (1) sponsorship (i.e., funding source), (2) number of participants enrolled (i.e., enrollment size), (3) date of submission (i.e., year of registration on ClinicalTrials.gov), (4) primary objective of the intervention, (5) number of sites, (6) geographic region of sites (i.e., involvement of European countries in the study), (7) location of the principal investigator’s office (i.e., country and continent), (8) study type and use of randomization, (9) longest follow-up duration for the primary outcome(s) (i.e., days), (10) recruitment status, and (11) publication status (i.e., published or unpublished trial). Minor adjustments were made to labels where necessary to ensure accuracy and clarity (e.g., trials with only a single arm were automatically labeled nonrandomized).

The National Library of Medicine describes the trial variable (1) “sponsorship” as organizations that provide funding and/or support in the form of study design, implementation, data analysis, or reporting [10]. “Industry” was classified as funding by any trial for which industry was listed as the lead or partnering agency. Non-industry trials with US federal agencies as a lead or partnering agency were classified as “government”. All remaining trials were predominantly funded by academic institutions or university- or community-based associations and were hence classified as “academic/university” or “non-profit”, respectively.

The trial characteristic (2) “number of participants enrolled” was further sub-classified into “small” and “large”, assuming *n* = 100 as the threshold number of patients to be enrolled to belong to either category.

In conducting an exploratory analysis, the approximate midpoint of the 239-month study period was selected and divided into a 76-month “early” period (1 January 2003 to 31 December 2009), a 76-month “interim” period (1 January 2010 to 31 December 2016) and a final 87-month “late” period (1 January 2017 to 31 December 2023).

The trial characteristic (4) “primary objective of the study” was retrieved from the ClinicalTrial.gov website and was further classified based on the type and aim of the intervention being tested as “procedures”, “devices”, or “drugs”.

The trial characteristics (5) “number of study sites” and (9) “longest follow-up duration for the primary outcome(s)” were further divided into “single-site” or “multi-site” for the former and “short” (0–30 days), “medium” (31–365 days), and “long” (>365 days) for the latter.

For the trial characteristic (10) “recruitment status”, any trial that was stopped was classified as “terminated”, “withdrawn”, or “suspended” as an early discontinuation. Trials that remained ongoing at the cutoff for analysis (31 December 2023) were excluded from the present analysis. Consequently, all of the other trials were assumed to have completed patient recruitment by the time of data extraction and were hence classified as “completed”.

The trial characteristic (11) “publication status” was assessed by searching on PubMed, Scopus, MEDLINE, or Google Scholar for published investigations incorporating the ClinicalTrials.gov ID or the study title to subsequently classify the trials as either “published” or “unpublished”.

### 2.3. Statistical Analysis

Statistical analyses were performed using R Statistical Software (version 4.1.1, Foundation for Statistical Computing, Vienna, Austria). Given that certain characteristics were either inapplicable or not reported for some trials, the total counts varied between dimensions accordingly. Data missing from calculations were excluded and not subject to imputation unless otherwise specified.

All year-to-year analyses included only years with a full 12-month collection of data (2003–2023).

Descriptive statistics were used to summarize trial data with frequencies and percentages.

For comparisons of continuous variables, the Mann–Whitney or Kruskal–Wallis test was employed. Trends over time were assessed using the Cochran–Mantel–Haenszel (CMH) test. Categorical variables underwent testing with either a Chi-square or a Fisher Exact test as deemed appropriate. To examine the correlation between the publication year and the annual number of registered trials on ClinicalTrials.gov, the Pearson correlation coefficient, R-squared (R^2^)—indicating the proportion of variance in the dependent variable explained by the independent variable—and the corresponding *p*-value was calculated, with significance defined as *p* < 0.05 for all analyses.

The data and study design do not permit the assessment of causality. The discussion of results was limited to studies that had both meaningful effect sizes and statistical significance, which was set at an α level of 0.05. Since exploratory analysis does not adjust for multiple comparisons, approximately 1 of every 100 tests was expected to produce a significant result due to chance.

## 3. Results

The search strategy yielded a total of 320 unique trial records on the ClinicalTrials.gov website, which constituted 0.07% of all contemporaneous trials registered on ClinicalTrials.gov.

Of these, *n* = 154 remained ongoing at the cutoff for analysis (31 December 2023), *n* = 3 focused on non-adult (i.e., pediatric) OHCA and *n* = 11 lacked clear information on the type of intervention being tested and were all excluded.

**Figure 2:**
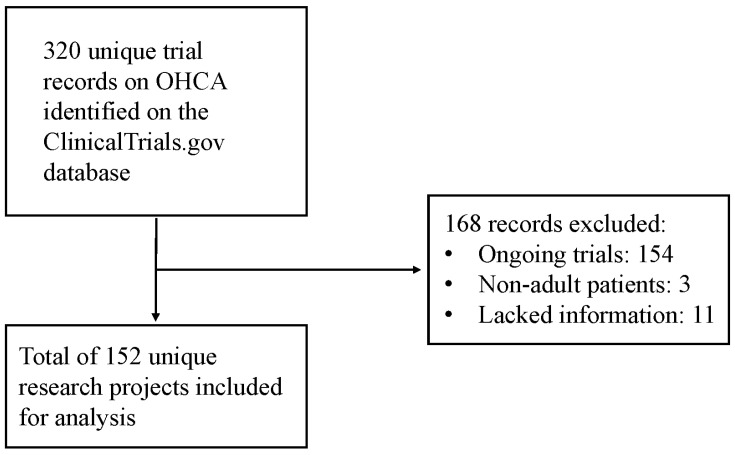
Flow diagram of the trial selection process.

Consequently, *n* = 152 trials were retrieved for inclusion in the present analysis (Figure 2).

### 3.1. General Characteristics of the OHCA Clinical Trials and Trends over Time

Out of *n* = 152 trials included in the present analysis, *n* = 45 (29.6%) were observational trials and *n* = 107 (70.4%) were interventional trials. Of the latter, 83% used randomization (Table 1).

Interestingly, notwithstanding the annual rate of OHCA trials registered on ClinicalTrials.gov over the study period (1 January 2003 to 31 December 2023) demonstrating a non-significant increase over time (correlation coefficient = 0.348, R^2^ = 0.121, *p* = 0.121), the number of trial submissions in our “late” period (37; 1 January 2017, to 31 December 2023) almost doubled the number of trial submissions in our “early” period (18; 1 January 2003 to 31 December 2009).

Furthermore, when being categorized into “early” (18; 1 January 2003 to 31 December 2009), “interim” (52; 1 January 2010 to 31 December 2016), and “late” (37; 1 January 2017 to 31 December 2023) periods, the interventional and observational trials exhibited statistically significant distinct patterns (*p* < 0.001).

By tracking the registration rate for clinical trials on the ClinicalTrials.gov website, we observed that the interventional and observational studies mainly differed in their primary focus. More specifically, the former were more often registered with “procedures” (43% vs. 38%), “devices” (23% vs. 20%), or “drugs” (21% vs. 0%) as their primary focus, and the latter as “biomarkers” (16% vs. 2%) or “diagnostic test” (13% vs. 4%) (*p* < 0.001).

Additionally, there was a trend suggesting that interventional studies may exhibit more likelihood of reaching full publication status compared to observational studies (66% vs. 51%; *p* = 0.113).

Furthermore, a greater proportion of observational studies included a mid-term follow-up period (31–365 days) compared to interventional studies (17.8% vs. 3.7%, *p* = 0.013).

Another discernible trend emerged within the studies registered in Europe, with observational studies exhibiting a higher prevalence compared to interventional studies (73% vs. 55%; *p* = 0.092). Moreover, 78% of observational studies and 58% of interventional studies identified Europe as the continent for the chairman’s office’s primary location. However, this trend did not achieve statistical significance (*p* = 0.104).

### 3.2. Characteristics of OHCA Interventional and Observational Trials over Time

In the analysis of *n* = 107 interventional and *n* = 45 observational trials registered on ClinicalTrials.gov, we systematically examined the temporal evolution of their distinctive attributes over the “early”, “interim”, and “late” periods spanning from 1 January 2003 to 31 December 2023.

The diverse characteristics of such trials (e.g., completion and publication status, use of randomization, primary focus, sample size and follow-up duration, funding source, number of participating sites and their location, etc.) throughout these defined temporal intervals were further scrutinized and synthesized, with a comprehensive summary provided in Table 2.

Significantly, there was a substantial decrement in the number of interventional trials with a sample size >100 patients across the three above-mentioned temporal study points (90%, 69%, 46%) in contrast to observational studies. In the latter, >50% consistently enrolled over 100 patients, regardless of the study period (*p* = 0.005). Furthermore, noteworthy patterns in follow-up duration were evident, revealing an extended median follow-up of 28 days for interventional studies in the “early” and “interim” periods in contrast to 3 days in the “late” period (*p* = 0.090).

The majority of interventional trials received predominant funding from the “academic/university” sector, and this financial backing demonstrated an increasing trajectory over time, escalating from 50% in the “early” period to over 85% in both the “interim” and “late” periods. In contrast, for observational studies, the “academic/university” sector remained the most frequent source of funding consistently across all three study periods, exceeding 70% (*p* = 0.044).

Furthermore, single-center participation was more common than multi-center participation in both interventional and observational studies across all three study periods. However, observational studies had a higher proportion of “N/A” studies compared to interventional studies during the “early” (20% vs. 6%) and “interim” (26% vs. 11.5%) periods (*p* = 0.030). As such, while the trend shows a consistent shift in both study types, more marked differences in site participation were seen in observational studies, particularly in the “late” period, where single-center participation increased, indicating a conspicuous inclination towards Europe as the favored location for participating centers (*p* = 0.083).

France consistently led as the primary location for the chairman’s office in observational studies across the “early” (40%), “interim” (21%), and “late” (14%) periods. However, for interventional studies, France held the leading position only during the “early” period (33%). In the “interim” period, the Republic of Korea was instead most frequently designated as the primary location for the chairman’s office in interventional studies (21%), while during the “late” period, Denmark, Sweden, and the United States each accounted for 16% (*p* = 0.035).

#### 3.2.1. Publication Characteristics

Table 3 summarizes the main characteristics of interventional (Table 3a) and observational (Table 3b) trials with respect to their completion and publication status. As of 31 December 2023, 36/107 (34%) OHCA interventional trials and 22/45 (49%) observational trials registered on ClinicalTrials.gov remained unpublished. Randomization was used in 83 of 107 interventional trials and in none of the observational trials, with a significant disparity between published and unpublished studies. Specifically, 84% of RCTs reached the full publication status compared with the 15% only of non-randomized trials (*p* = 0.03).

Interestingly, interventional trials with larger sample sizes exhibited a higher likelihood of being published compared to those with smaller sample sizes (median of 336 vs. 73 patients; *p* = 0.003). Published interventional studies predominantly had a sample size exceeding 100 patients (76%), in contrast to unpublished trials (*p* < 0.001). Additionally, a discernible trend suggested a delayed registration timeline among unpublished interventional trials (median 2016 [2012, 2019]) compared to published ones (median 2015 [2010, 2017]; *p* = 0.073).

Furthermore, there was a noteworthy trend indicating that multicenter interventional trials were more frequently published (41% vs. 19%) compared to single-center studies (55% vs. 69%) (*p* = 0.054). A significant difference was observed in the number of sites involved (*p* = 0.039), with a higher proportion of published interventional trials conducted in European centers (62%), while the majority of unpublished studies were conducted outside of Europe (56%) (*p* = 0.012).

Examining details by country, the United States served as the primary location for the chairman’s office in 21% of published interventional trials, followed by France at 14%, whereas the Republic of Korea was the primary location for 28% of unpublished studies (*p* = 0.055). Almost 60% of unpublished interventional studies and 42% of published studies encompassed Europe and Asia, respectively, as the continent of the chairman’s office’s primary location (*p* < 0.001). Notably, the only significant difference between published and unpublished observational trials was the primary location of the chairman’s office (*p* = 0.02). Specifically, France was the primary location for the majority of unpublished observational studies (32%), while Slovenia led among published studies (17%).

#### 3.2.2. Comparison of Single- vs. Multi-Site Study Characteristics

Table 4 summarizes the characteristics of single- (*n* = 64) and multi-center (*n* = 36) interventional studies (Table 4a) and of single- (*n* = 29) and multi-center (*n* = 10) observational studies (Table 4b). Seven OHCA interventional trials and six OHCA observational trials registered on the ClinicalTrials.gov website did not report information on site locations and were hence excluded from the present analysis.

Interestingly, RCTs exhibited a tendency favoring multi-center interventional trials over single-center trials (89% vs. 72%; *p* = 0.136).

Nevertheless, a trend was observed towards a higher proportion of single-center interventional trials with a planned sample size >100 patients compared to multi-center studies (60% vs. 43%; *p* = 0.085). As such, this trend was not mirrored among observational studies.

The median number of participating centers in multi-center interventional studies was 6 [2,3,4,5,6,7,8,9,10] (*p* < 0.001) and 5.50 [2.00, 7.75] (*p* < 0.001) for observational trials.

An additional distinction between observational and interventional trials, based on the number of centers involved (i.e., single-site vs. multi-site), was observed in trial completion status. Specifically, 100% of observational studies were fully completed, compared to only 80% of interventional studies (*p* < 0.026).

Geographically, half (50%) of the multi-center interventional trials and 60% of the single-center trials were conducted in Europe (*p* < 0.001).

The United States served as the primary location for the chairman’s office in one-third of multi-center interventional studies, while France led in single-center studies (20%) (*p* = 0.023).

When considering Europe as a whole, it emerged as the continent with a higher prevalence of both multi-center and single-center interventional studies (58% and 59%, respectively), in comparison to North America, Asia, and Oceania (*p* < 0.006).

## 4. Discussion

The present investigation sought to provide a comprehensive overview of the OHCA-related clinical trials registered in the ClinicalTrials.gov database. To the best of the authors’ knowledge, it represents the first publicly available extensive assessment of the characteristics of OHCA-related clinical trials.

Our results show that clinical trials on OHCA were mostly interventional and randomized. Interestingly, the overall proportion of interventional trials (70.4%) mirrored that of other disease trials [11,12], being predominantly treatment-oriented and focused on “procedures”, “devices”, or “drugs”.

As evidence-based medicine continues to evolve, meticulously designed RCTs hold a significant role in shaping health policies and informing clinical decisions. As such, the absence of randomization significantly heightens the risk of bias in trial outcomes [13]. Moreover, randomization is imperative for establishing causal relationships between treatment and outcome, as well as for evaluating treatment cost-effectiveness. In contrast, non-randomized trials cannot conclusively rule out the potential influence of a third factor on observed associations [14]. Accordingly, our study showed that 83% of the interventional trials were randomized, and thus potentially capable of generating novel, high-quality evidence.

In contrast, observational studies in the field of OHCA were more likely to be completed than interventional trials, even when they involved a multi-center design and larger sample sizes (≥100 patients). However, the higher proportion of observational studies where the single- or multi-center design was not clearly specified in the data provided by authors on the ClinicalTrials.gov repository raised potential additional concerns about study quality. Such an omission highlights the need for improved study design and data reporting in observational trials, as these factors can—albeit partially—influence the overall quality and reliability of the findings.

In terms of follow-up, the majority of interventional and observational studies included a 365-day follow-up period. Short-term (0–30 days) to mid-term (31–365 days) follow-ups were likely implemented to address pragmatic needs, reflecting the authors’ assumption that a single intervention is unlikely to have sustained effects beyond this period. However, pre-specifying longer follow-up durations could still provide valuable insights into the long-term impact of the interventions. As such, we believe that future research should consider incorporating comprehensive assessments of quality of life in OHCA survivors over extended follow-up intervals.

The temporal evolution of trial attributes was analyzed across the “early”, “interim”, and “late” periods from 1 January 2003 to 31 December 2023 as well. The diverse characteristics of these trials likely reflected the expertise and resources of high-income countries (e.g., France, Denmark, Sweden, USA), where the significant social and economic burden of OHCA is well recognized. These nations may have a greater capacity to initiate and conduct trials in this area. This assumption is further supported by the observation inferred from our investigation that the primary location of the chairman’s office frequently resided in high-income countries.

On the other hand, it is possible that variations in study quality—such as shortcomings in study design, deficiencies in trial management, negative or unexpected results, or unforeseen biases and misinformation—may contribute to the early termination of clinical trials or their failure to be published. These quality disparities highlight a crucial dimension that warrants thorough evaluation within the context of registered OHCA clinical trials.

Furthermore, our analysis revealed a consistent increase in the number of interventional and observational studies funded by the “academic/university” sector, conducted at a single center and featuring a small sample size (<100) across the study intervals spanning 2003–2009, 2010–2016, and 2017–2023. OHCA, being a significant public health concern, requires a coordinated effort from the entire community for its effective management. Given its time-sensitive nature, there is a growing consensus on the necessity of greater involvement of community hospitals in addressing this pressing issue. These hospitals, typically located in more peripheral areas, often experience a higher influx of patients, rendering them crucial focal points for OHCA management strategies. Based on the findings of our analysis, it is strongly recommended that future initiatives prioritize the integration of community hospitals into wider OHCA response frameworks. Such an enhanced collaboration shows potential to improve the accessibility of life-saving interventions and strengthen the overall resilience of our healthcare system against this critical and life-threatening condition. Nevertheless, in contrast to certain clinical settings (e.g., oncology), where increasing agreement that small-scale clinical trials focusing on either genetics or biomarkers can deliver conclusive findings is being reached [11], it can be hypothesized that within the wider acute care setting, such trials might not adequately address certain treatment dimensions (e.g., evaluating therapies with moderate effects) [11,15]. Thus, in the context of OHCA, only larger-scale clinical trials may prove to be essential to holistically address existing clinical challenges. Notwithstanding, we observed an increase in the number of small-scale interventional trials throughout the three specified study periods. This trend could be attributed in part to the increasing financial constraints faced by researchers or clinicians, which may restrict participant recruitment rates for trials. Additionally, another contributing factor could be that small-sample trials were less frequently registered on ClinicalTrials.gov in earlier years but became strongly recommended in later years due to a changed trial publication policy. This notion is further supported by our findings, which indicate a non-significant increase over time in trial submissions during the “late” period, nearly doubling the number of trial submissions compared to the “early” period. This also mirrors the notable increase in the registration rate of clinical trials observed following the guideline proposed by the International Committee of Medical Journal Editors (ICMJE), which advocates for the registration of clinical trials in a public registry prior to participant recruitment [16,17].

We also estimated that most interventional studies throughout the three study periods were carried out at a single center. This could be attributed to potential significant variations in health policies among different regions, which might hinder the collaboration among staff and administration in multiple centers and ultimately restrict the feasibility of conducting multi-center trials. Future studies should address this constraint, given the significance of OHCA in both healthcare and human expenses.

Interestingly, we observed that interventional trials with a large patient sample size (>100) were more likely to be published. As a result, we found that studies registered on ClinicalTrials.gov had a greater likelihood of being published, with interventional trials reaching a publication rate of 66%.

Similarly, our observations suggest a prevailing tendency to plan a greater number of non-randomized single-center trials with a targeted sample size exceeding 100 patients, in contrast to multi-center studies. Although this trend may appear contradictory, increasing the sample size in a single non-randomized study does not always guarantee conclusive results. Therefore, this observation indicates a significant deviation from conventional clinical trial planning expectations and highlights the scarcity of multi-center RCTs with >100 patients. Hence, it is vital for future studies to aim for this benchmark. Moreover, this acknowledgment emphasizes the necessity of adjusting trial methodologies to ensure the attainment of robust and applicable findings. We calculated that despite the noted increase in trial registration rates, close to 35% of completed interventional studies remained unpublished, thus restricting access to outcomes. This rate of non-publication for completed studies aligns with findings in other medical domains, indicating the necessity for enhanced oversight in publishing OHCA articles [18]. Ensuring the timely dissemination of trial results is crucial for generating the strongest scientific evidence and maximizing benefits for both public health and scientific advancement [19]. Interestingly, reasons for the non-publication of completed interventional trials might encompass negative results that contradict the researcher’s hypothesis and editors’ preferences for positive findings, making negative results less likely to be published [18,20,21].

A final significant remark emphasized in our report was the increase in funding observed throughout the three study periods analyzed, particularly from the “academic/university” sector. This trend suggests potential benefits, as it could indirectly enhance the precision and continual oversight during the execution of clinical trials. Conversely, funding from alternative sources (e.g., industry sponsors) may enable larger, multicenter trials with extended durations, yet it also introduces concerns regarding the thoroughness and impartiality of monitoring, along with challenges related to data management, reporting accuracy, and the possibility of selective publication bias. Such challenges have been documented in prior industry-funded trials across diverse clinical settings [22,23].

### Study Limitations and Future Perspectives

Our research suffered several limitations. First, due to the option for investigators and sponsors to register trials in global registries other than ClinicalTrials.gov to meet the registration-before-enrollment criterion of the Committee of Medical Journal Editors, our approach lacked a systematic framework [24].

Additionally, since the data for all clinical trials on ClinicalTrials.gov were sourced from investigator reports, and not all data on the platform are validated by the US National Library of Medicine, we could not consistently guarantee the accuracy of all trial information [24].

Moreover, our extensive search might have overlooked smaller, unfunded trials, which are less likely to be registered, particularly if they lack significant findings and their results are never published, potentially introducing selection bias into our findings. Additionally, the completeness and currency of the datasets for all clinical trials in the database cannot always be guaranteed (e.g., some studies may have been missed during the selection process, leading to potential misclassifications).

Another limitation is that the database we created may not have captured all published literature, especially manuscripts not uploaded to ClinicalTrials.gov or indexed in PubMed, as well as abstracts or manuscripts currently under review, revision, or recently accepted. Lastly, it is worth noting that the requirements for using ClinicalTrials.gov have changed over the past decade, and retrospective posting was not mandatory. Despite these limitations, ClinicalTrials.gov remains a valuable data source, encompassing over 70% of all clinical trials in the World Health Organization International Clinical Trials Registry [25].

In order to enhance the credibility of this website and broaden access to health information for all members of the OHCA research community, we propose that investigators familiarize themselves with the regulations mandating the registration of OHCA trials, regularly update their trial results, and upload publications to the database. Similarly, funding agencies should recognize that this is an ongoing responsibility and allocate ongoing resources to meet these requirements. Additionally, we suggest that in order to enhance the transparency of OHCA research conduct and reporting, there is a need for further global standardization of rules for clinical trial registries [26].

## 5. Conclusions

Our study comprised the first systematic report outlining the characteristics and publication status of all OHCA clinical trials registered on ClinicalTrials.gov. It serves as a dependable foundation for evidence-based medicine, aids in shaping health policies, and shows potential to foster advancements in public health and clinical medicine [19]. Our findings revealed that clinical trials on OHCA primarily involved interventions aimed at treatment and were often randomized. Additionally, we observed a consistent increase in interventional studies funded by the “academic/university” sector, conducted at single centers, and featuring small sample sizes (<100) across the study periods from 2003 to 2023. Conversely, interventional trials registered on ClinicalTrials.gov with larger patient sample sizes (>100) had a higher probability of publication, reaching 66%. To enhance the transparency of OHCA clinical trials, researchers should adhere to standardized registration procedures and promptly publish positive and negative clinical findings alike.

## Figures and Tables

**Table 1:** Characteristics of interventional vs. observational trials.

	Overall	Interventional	Observational	*p*-Value
	(N = 152)	(N = 107)	(N = 45)	
Status, *n* (%)				0.248
Completed	137 (90.1)	94 (87.9)	43 (95.6)	
Terminated	15 (9.9)	13 (12.1)	2 (4.4)	
Randomized trial, *n* (%)				
No	69 (45.4)	24 (22.4)	45 (100.0)	<0.001
Yes	83 (54.6)	83 (77.6)	0 (0.0)	
Availability of results, *n* (%) *				0.006
No	128 (84.2)	84 (78.5)	44 (97.8)	
Yes	24 (15.8)	23 (21.5)	1 (2.2)	
Publication status, *n* (%) †				0.113
No	58 (38.2)	36 (33.6)	22 (48.9)	
Yes	94 (61.8)	71 (66.4)	23 (51.1)	
Primary focus, *n* (%)				<0.001
Procedures	63 (41.4)	46 (43.0)	17 (37.8)	
Devices	35 (23.0)	26 (24.3)	9 (20.0)	
Drugs	22 (14.5)	22 (20.6)	0 (0.0)	
Diagnostic	10 (6.6)	4 (3.7)	6 (13.3)	
Biomarkers	9 (5.9)	2 (1.9)	7 (15.6)	
Other	13 (8.6)	7 (6.5)	6 (13.3)	
Median enrollment size [IQR], *n*	150 [57.75, 724]	200 [60, 801]	112 [50, 460]	0.235
Planned sample size ≥100 patients, *n* (%)				0.551
Yes	95 (62.5)	69 (64.5)	26 (57.8)	
No	57 (37.5)	38 (35.5)	19 (42.2)	
Median follow-up [IQR], days	10 [0, 180]	12 [0, 180]	5 [0, 210]	0.547
Follow-up categories, *n* (%)				0.013
0–30 days	116 (76.3)	85 (79.4)	31 (68.9)	
31–365 days	12 (7.9)	4 (3.7)	8 (17.8)	
>365 days	24 (15.8)	18 (16.8)	6 (13.3)	
Funder type, *n* (%)				0.651
Academic/university	122 (80.3)	87 (81.3)	35 (77.8)	
Government	18 (11.8)	11 (10.3)	7 (15.6)	
Industry	10 (6.6)	7 (6.5)	3 (6.7)	
No-profit	2 (1.3)	2 (1.9)	0 (0.0)	
Median start date [IQR], year	2016 [2012, 2018]	2015 [2011, 2017.50]	2016 [2013, 2018]	0.150
Study period, *n* (%)				0.338
Early	23 (15.1)	18 (16.8)	5 (11.1)	
Interim	71 (46.7)	52 (48.6)	19 (42.2)	
Late	58 (38.2)	37 (34.6)	21 (46.7)	
Median number of sites involved [IQR], *n*	1 [1, 2]	1 [1, 2.25]	1 [1, 1.50]	0.209
Participating sites, *n* (%)				0.203
Single-center	93 (61.2)	64 (59.8)	29 (64.4)	
Multi-center	46 (30.3)	36 (33.6)	10 (22.2)	
N/A	13 (8.6)	7 (6.5)	6 (13.3)	
Site location, *n* (%)				0.092
Europe	92 (60.5)	59 (55.1)	33 (73.3)	
Outside of Europe	52 (34.2)	41 (38.3)	11 (24.4)	
Both within and outside of Europe	6 (3.9)	6 (5.6)	0 (0.0)	
N/A	2 (1.3)	1 (0.9)	1 (2.2)	
Chairman’s office—primary location (country-detailed), *n* (%)				0.272
France	25 (16.4)	16 (15.0)	9 (20.0)	
United States	24 (15.8)	20 (18.7)	4 (8.9)	
Korea, Republic of	18 (11.8)	14 (13.1)	4 (8.9)	
Sweden	16 (10.5)	12 (11.2)	4 (8.9)	
Denmark	11 (7.2)	8 (7.5)	3 (6.7)	
Norway	6 (3.9)	4 (3.7)	2 (4.4)	
Slovenia	6 (3.9)	2 (1.9)	4 (8.9)	
Taiwan	6 (3.9)	5 (4.7)	1 (2.2)	
Canada	5 (3.3)	4 (3.7)	1 (2.2)	
Finland	4 (2.6)	2 (1.9)	2 (4.4)	
Germany	4 (2.6)	3 (2.8)	1 (2.2)	
Spain	4 (2.6)	4 (3.7)	0 (0.0)	
Netherlands	4 (2.6)	3 (2.8)	1 (2.2)	
Italy	3 (2.0)	1 (0.9)	2 (4.4)	
United Kingdom	3 (2.0)	0 (0.0)	3 (6.7)	
Australia	2 (1.3)	2 (1.9)	0 (0.0)	
Belgium	2 (1.3)	2 (1.9)	0 (0.0)	
Other	9 (5.9)	5 (5.7)	4 (8.9)	
Chairman’s office—primary location (continent) (%)				0.104
Europe	97 (63.8)	62 (57.9)	35 (77.8)	
North America	27 (17.8)	23 (21.5)	4 (8.9)	
Asia	26 (17.1)	20 (18.7)	6 (13.3)	
Oceania	2 (1.3)	2 (1.9)	0 (0.0)	

* Refers to the summary of the results reported in the ClinicalTrials.gov database. Summary results information includes participant flow, baseline characteristics, outcome measures, and adverse events. † Refers to the achievement of trial’s full publication status. Percentages may not total 100 because of rounding. IQR denotes interquartile range.

**Table 2:** Characteristics of interventional and observational trials over time.

	Early Period	Interim Period	Late Period	Early Period	Interim Period	Late Period	*p*-Value
	(N = 18)	(N = 52)	(N = 37)	(N = 5)	(N = 19)	(N = 21)	
	**Interventional Trials**			**Observational Trials**			
Trial status, *n* (%)							0.337
Completed	16 (88.9)	47 (90.4)	31 (83.8)	5 (100)	19 (100)	19 (90.5)	
Terminated	2 (11.1)	5 (9.6)	6 (16.2)	0 (0)	0 (0)	2 (9.5)	
Randomized trial, *n* (%)							
No	3 (16.7)	13 (25.0)	8 (21.6)	5 (100.0)	19 (100.0)	21 (100.0)	0.759
Yes	15 (83.3)	39 (75.0)	29 (78.4)	0 (0.0)	0 (0.0)	0 (0.0)	
Published as of 31 December 2023, *n* (%)							0.363
No	3 (16.7)	18 (34.6)	15 (40.5)	3 (60.0)	8 (42.1)	11 (52.4)	
Yes	15 (83.3)	34 (65.4)	22 (59.5)	2 (40.0)	11 (57.9)	10 (47.6)	
Trial status and publication, *n* (%)							0.594
Completed and published	13 (72.2)	31 (59.6)	18 (48.6)	2 (40.0)	11 (57.9)	10 (47.6)	
Terminated and published	2 (11.1)	3 (5.8)	4 (10.8)	0 (0.0)	0 (0.0)	0 (0.0)	
Completed and unpublished	3 (16.7)	16 (30.8)	13 (35.1)	3 (60.0)	8 (42.1)	9 (42.9)	
Uncompleted and unpublished	0 (0.0)	2 (3.8)	2 (5.4)	0 (0.0)	0 (0.0)	2 (9.5)	
Availability of results, *n* (%)							0.474
No	14 (77.8)	39 (75.0)	31 (83.8)	5 (100.0)	18 (94.7)	21 (100.0)	
Yes	4 (22.2)	13 (25.0)	6 (16.2)	0 (0.0)	1 (5.3)	0 (0.0)	
Primary focus, *n* (%)							0.256
Procedures	7 (38.9)	28 (53.8)	11 (29.7)	1 (20.0)	7 (36.8)	9 (42.9)	
Devices	5 (27.8)	9 (17.3)	12 (32.4)	1 (20.0)	4 (21.1)	4 (19.0)	
Drugs	5 (27.8)	9 (17.3)	8 (21.6)	0 (0.0)	0 (0.0)	0 (0.0)	
Biomarkers	1 (5.6)	1 (1.9)	0 (0.0)	2 (40.0)	3 (15.8)	2 (9.5)	
Diagnostic test	0 (0.0)	1 (1.9)	3 (8.1)	0 (0.0)	2 (10.5)	4 (19)	
Other	0 (0.0)	4 (7.7)	3 (8.1)	1 (20.0)	3 (15.8)	2 (9.5)	
Median enrollment size [IQR], *n*	685 [299.25, 1579.50]	238.50 [85.50, 625]	65 [34, 397]	1662 [350, 2332]	112 [40.5, 232]	100 [50, 460]	0.495
Planned sample size ≥100 patients, *n* (%)							0.005
No	2 (11.1)	16 (30.8)	20 (54.1)	1 (20.0)	8 (42.1)	10 (47.6)	
Yes	16 (88.9)	36 (69.2)	17 (45.9)	4 (80.0)	11 (57.9)	11 (52.4)	
Median follow-up duration [IQR], days	28 [1, 90]	28 [0.50, 180]	3 [0, 105]	4 [2.5, 184.5]	5 [0, 180]	28 [0, 365]	0.099
Follow-up categories, *n* (%)							0.740
0–30 days	13 (72.2)	42 (80.8)	30 (81.1)	1 (20.0)	15 (78.9)	15 (71.4)	
31–365 days	0 (0.0)	2 (3.8)	2 (5.4)	1 (20.0)	2 (10.5)	5 (23.8)	
>365 days	5 (27.8)	8 (15.4)	5 (13.5)	3 (60.0)	2 (10.5)	1 (4.8)	
Funder type, *n* (%)							0.044
Academic/university	9 (50.0)	46 (88.5)	32 (86.5)	5 (100.0)	15 (78.9)	15 (71.4)	
Government	5 (27.8)	2 (3.8)	4 (10.8)	0 (0.0)	2 (10.5)	5 (23.8)	
Industry	4 (22.2)	3 (5.8)	0 (0.0)	0 (0.0)	2 (10.5)	1 (4.8)	
Non-profit	0 (0.0)	1 (1.9)	1 (2.7)	0 (0.0)	0 (0.0)	0 (0.0)	
Median number of sites [IQR], *n*	1 [1, 5]	1 [1, 3]	1 [1, 2]	1 [1, 2.5]	1 [1, 1.75]	1 [1, 1]	0.530
Number of participating sites, *n* (%)							0.030
Single-center	11 (61.1)	29 (55.8)	24 (64.9)	3 (60.0)	10 (52.6)	16 (76.2)	
Multi-center	6 (33.3)	17 (32.7)	13 (35.1)	1 (20.0)	4 (21.1)	5 (23.8)	
N/A	1 (5.6)	6 (11.5)	0 (0.0)	1 (20.0)	5 (26.3)	0 (0.0)	
Participating center’s location, *n* (%)							0.083
Europe	11 (61.1)	24 (46.2)	24 (64.9)	4 (80.0)	13 (68.4)	16 (76.2)	
Outside of Europe	4 (22.2)	25 (48.1)	12 (32.4)	1 (20.0)	5 (26.3)	5 (23.8)	
Both within and outside of Europe	3 (16.7)	2 (3.8)	1 (2.7)	0 (0.0)	0 (0.0)	0 (0.0)	
N/A	0 (0.0)	1 (1.9)	0 (0.0)	0 (0.0)	1 (5.3)	0 (0.0)	
Chairman’s office—primary location (country-detailed), *n* (%)							0.035
France	6 (33.3)	6 (11.5)	4 (10.8)	2 (40.0)	4 (21.1)	3 (14.3)	
United States	5 (27.8)	9 (17.3)	6 (16.2)	0 (0.0)	2 (10.5)	2 (9.5)	
Korea, Republic of	0 (0.0)	11 (21.2)	3 (8.1)	0 (0.0)	2 (10.5)	2 (9.5)	
Sweden	0 (0.0)	6 (11.5)	6 (16.2)	0 (0.0)	1 (5.3)	3 (14.3)	
Denmark	0 (0.0)	2 (3.8)	6 (16.2)	0 (0.0)	1 (5.3)	2 (9.5)	
Norway	0 (0.0)	2 (3.8)	2 (5.4)	0 (0.0)	1 (5.3)	1 (4.8)	
Slovenia	0 (0.0)	1 (1.9)	1 (2.7)	0 (0.0)	2 (10.5)	2 (9.5)	
Taiwan	2 (11.1)	1 (1.9)	2 (5.4)	0 (0.0)	0 (0.0)	1 (4.8)	
Canada	0 (0.0)	3 (5.8)	1 (2.7)	1 (20.0)	0 (0.0)	0 (0.0)	
Finland	1 (5.6)	1 (1.9)	0 (0.0)	0 (0.0)	1 (5.3)	1 (4.8)	
Germany	2 (11.1)	1 (1.9)	0 (0.0)	1 (20.0)	0 (0.0)	0 (0.0)	
Spain	1 (5.6)	3 (5.8)	0 (0.0)	0 (0.0)	0 (0.0)	0 (0.0)	
Netherlands	0 (0.0)	1 (1.9)	2 (5.4)	0 (0.0)	1 (5.3)	0 (0.0)	
Italy	0 (0.0)	0 (0.0)	1 (2.7)	0 (0.0)	2 (10.5)	0 (0.0)	
United Kingdom	0 (0.0)	0 (0.0)	0 (0.0)	0 (0.0)	0 (0.0)	3 (14.3)	
Australia	0 (0.0)	2 (3.8)	0 (0.0)	0 (0.0)	0 (0.0)	0 (0.0)	
Belgium	1 (5.6)	0 (0.0)	1 (2.7)	0 (0.0)	0 (0.0)	0 (0.0)	
Other	0 (0.0)	3 (5.8)	2 (5.4)	1 (20)	2 (10.5)	1 (4.8)	
Chairman’s office—primary location (continent), *n* (%)							0.351
Europe	11 (61.1)	25 (48.1)	26 (70.3)	5 (100.0)	14 (73.7)	16 (76.2)	
North America	5 (27.8)	12 (23.1)	6 (16.2)	0 (0.0)	2 (10.5)	2 (9.5)	
Asia	2 (11.1)	13 (25.0)	5 (13.5)	0 (0.0)	3 (15.8)	3 (14.3)	
Oceania	0 (0.0)	2 (3.8)	0 (0.0)	0 (0.0)	0 (0.0)	0 (0.0)	

Percentages may not total 100 because of rounding. IQR denotes interquartile range. N/A, not available.

**Table 3:** Characteristics of unpublished vs. published (**a**) interventional and (**b**) observational trials.

(a)				
	Overall	Unpublished	Published	*p*-Value
	(N = 107)	(N = 36)	(N = 71)	
Trial status, *n* (%)				1.00
Completed	94 (87.9)	32 (88.9)	62 (87.3)	
Terminated	13 (12.1)	4 (11.1)	9 (12.7)	
Randomized trial, *n* (%)				0.030
No	24 (22.4)	13 (36.1)	11 (15.5)	
Yes	83 (77.6)	23 (63.9)	60 (84.5)	
Availability of results, *n* (%)				0.906
No	84 (78.5)	29 (80.6)	55 (77.5)	
Yes	23 (21.5)	7 (19.4)	16 (22.5)	
Primary focus, *n* (%)				0.310
Procedures	46 (43.0)	15 (41.7)	31 (43.7)	
Devices	26 (24.3)	12 (33.3)	14 (19.7)	
Drugs	22 (20.6)	4 (11.1)	18 (25.4)	
Diagnostic test	4 (3.7)	2 (5.6)	2 (2.8)	
Biomarkers	2 (1.9)	0 (0.0)	2 (2.8)	
Other	7 (6.5)	3 (8.3)	4 (5.6)	
Median enrollment size [IQR], *n*	200 [60, 801]	73 [46, 544]	336 [100, 1000]	0.003
Planned sample size ≥100 patients, *n* (%)				<0.001
No	38 (35.5)	21 (58.3)	17 (23.9)	
Yes	69 (64.5)	15 (41.7)	54 (76.1)	
Median follow-up duration [IQR], days	12 [0, 180]	1.50 [0, 112.50]	28 [1, 180]	0.192
Follow-up categories, *n* (%)				0.801
0–30 days	85 (79.4)	29 (80.6)	56 (78.9)	
31–365 days	4 (3.7)	3 (8.3)	1 (1.4)	
>365 days	18 (16.8)	4 (11.1)	14 (19.7)	
Funder type, *n* (%)				0.648
Academic/university	87 (81.3)	31 (86.1)	56 (78.9)	
Government	11 (10.3)	2 (5.6)	9 (12.7)	
Industry	7 (6.5)	2 (5.6)	5 (7.0)	
Non-profit	2 (1.9)	1 (2.8)	1 (1.4)	
Median starting date [IQR], year	2015 [2011, 2017.50]	2016 [2012, 2019]	2015 [2010, 2017]	0.073
Study period, *n* (%)				0.209
early	18 (16.8)	3 (8.3)	15 (21.1)	
interim	52 (48.6)	18 (50.0)	34 (47.9)	
late	37 (34.6)	15 (41.7)	22 (31.0)	
Median number of sites [IQR], *n*	1 [1, 2.25]	1 [1, 1]	1 [1, 5.25]	0.039
Number of participating sites, *n* (%)				0.054
Single-center	64 (59.8)	25 (69.4)	39 (54.9)	
Multi-center	36 (33.6)	7 (19.4)	29 (40.8)	
N/A	7 (6.5)	4 (11.1)	3 (4.2)	
Participating center’s location, n (%)				0.012
Europe only	59 (55.1)	15 (41.7)	44 (62.0)	
Outside of Europe only	41 (38.3)	20 (55.6)	21 (29.6)	
Both within and outside of Europe	6 (5.6)	0 (0.0)	6 (8.5)	
N/A	1 (0.9)	1 (2.8)	0 (0.0)	
Chairman’s office—primary location (country-detailed), *n* (%)				0.055
France	16 (15.0)	6 (16.7)	10 (14.1)	
United States	20 (18.7)	5 (13.9)	15 (21.1)	
Korea, Republic of	14 (13.1)	10 (27.8)	4 (5.6)	
Sweden	12 (11.2)	3 (8.3)	9 (12.7)	
Denmark	8 (7.5)	1 (2.8)	7 (9.9)	
Norway	4 (3.7)	2 (5.6)	2 (2.8)	
Slovenia	2 (1.9)	0 (0.0)	2 (2.8)	
Taiwan	5 (4.7)	4 (11.1)	1 (1.4)	
Canada	4 (3.7)	1 (2.8)	3 (4.2)	
Finland	2 (1.9)	0 (0.0)	2 (2.8)	
Germany	3 (2.8)	0 (0.0)	3 (4.2)	
Spain	4 (3.7)	0 (0.0)	4 (5.6)	
Netherlands	3 (2.8)	1 (2.8)	2 (2.8)	
Italy	1 (0.9)	0 (0.0)	1 (1.4)	
United Kingdom	0 (0.0)	0 (0.0)	0 (0.0)	
Australia	2 (1.9)	0 (0.0)	2 (2.8)	
Belgium	2 (1.9)	1 (2.8)	1 (1.4)	
Other	5 (4.7)	2 (5.6)	3 (4.2)	
Chairman’s office—primary location (continent), *n* (%)				<0.001
Europe	62 (57.9)	15 (41.7)	46 (64.8)	
North America	23 (21.5)	6 (16.7)	18 (25.4)	
Asia	20 (18.7)	15 (41.7)	5 (7.0)	
Oceania	2 (1.9)	0 (0.0)	2 (2.8)	
**(b)**				
	**Overall**	**Unpublished**	**Published**	***p*** **-Value**
	**(N = 45)**	**(N = 22)**	**(N = 23)**	
Trial status, *n* (%)				
Completed	43 (95.6)	20 (90.9)	23 (100.0)	0.450
Terminated	2 (4.4)	2 (9.1)	0 (0.0)	
Availability of results, *n* (%)				
No	44 (97.8)	22 (100.0)	22 (95.7)	1.000
Yes	1 (2.2)	0 (0.0)	1 (4.3)	
Primary focus, *n* (%)				
Procedures	17 (37.8)	7 (31.8)	10 (43.5)	0.746
Devices	9 (20.0)	5 (22.7)	4 (17.4)	
Drugs	0 (0.0)	0 (0.0)	0 (0.0)	
Diagnostic test	6 (13.3)	2 (9.1)	4 (17.4)	
Biomarkers	7 (15.6)	4 (18.2)	3 (13.0)	
Other	6 (13.3)	4 (18.2)	2 (8.7)	
Median enrollment size [IQR], *n*	112 [50, 460]	106.50 [56.50, 553]	150 [50, 282]	0.856
Planned sample size ≥100 patients, *n* (%)				0.899
No	19 (42.2)	10 (45.5)	9 (39.1)	
Yes	26 (57.8)	12 (54.5)	14 (60.9)	
Median follow-up duration [IQR], days	5 [0, 210]	30 [0, 365]	2 [0.25, 142.50]	0.400
Follow-up categories, *n* (%)				0.248
0–30 days	31 (68.9)	13 (59.1)	18 (78.3)	
31–365 days	8 (17.8)	6 (27.3)	2 (8.7)	
>365 days	6 (13.3)	3 (13.6)	3 (13.0)	
Funder type, *n* (%)				0.552
Academic/university	35 (77.8)	18 (81.8)	17 (73.9)	
Government	7 (15.6)	2 (9.1)	5 (21.7)	
Industry	3 (6.7)	2 (9.1)	1 (4.3)	
Non-profit	0 (0.0)	0 (0.0)	0 (0.0)	
Median starting date [IQR], year	2016 [2013, 2018]	2016.50 [2012.25, 2018.75]	2016 [2015, 2018]	0.900
Study period, *n* (%)				0.705
Early	5 (11.1)	3 (13.6)	2 (8.7)	
Interim	19 (42.2)	8 (36.4)	11 (47.8)	
Late	21 (46.7)	11 (50.0)	10 (43.5)	
Median number of sites [IQR], *n*	1 [1, 1.50]	1 [1, 2]	1 [1, 1]	0.840
Number of participating sites, *n* (%)				0.602
Single-center	29 (64.4)	14 (63.6)	15 (65.2)	
Multi-center	10 (22.2)	6 (27.3)	4 (17.4)	
N/A	6 (13.3)	2 (9.1)	4 (17.4)	
Participating center’s location, *n* (%)				1.000
Europe only	33 (73.3)	16 (72.7)	17 (73.9)	
Outside of Europe only	11 (24.4)	6 (27.3)	5 (21.7)	
Both within and outside of Europe	0 (0.0)	0 (0.0)	0 (0.0)	
N/A	1 (2.2)	0 (0.0)	1 (4.3)	
Chairman’s office—primary location (country-detailed), *n* (%)				0.020
France	9 (20.0)	7 (31.8)	2 (8.7)	
United States	4 (8.9)	1 (4.5)	3 (13.0)	
Korea, Republic of	4 (8.9)	4 (18.2)	0 (0.0)	
Sweden	4 (8.9)	1 (4.5)	3 (13.0)	
Denmark	3 (6.7)	1 (4.5)	2 (8.7)	
Norway	2 (4.4)	1 (4.5)	1 (4.3)	
Slovenia	4 (8.9)	0 (0.0)	4 (17.4)	
Taiwan	1 (2.2)	1 (4.5)	0 (0.0)	
Canada	1 (2.2)	0 (0.0)	1 (4.3)	
Finland	2 (4.4)	2 (9.1)	0 (0.0)	
Germany	1 (2.2)	0 (0.0)	1 (4.3)	
Spain	0 (0.0)	0 (0.0)	0 (0.0)	
Netherlands	1 (2.2)	0 (0.0)	1 (4.3)	
Italy	2 (4.4)	0 (0.0)	2 (8.7)	
United Kingdom	3 (6.7)	1 (4.5)	2 (8.7)	
Australia	0 (0.0)	0 (0.0)	0 (0.0)	
Belgium	0 (0.0)	0 (0.0)	0 (0.0)	
Other	4 (8.9)	3 (13.5)	1 (4.3)	
Chairman’s office—primary location (continent), *n* (%)				0.187
Europe	35 (77.8)	16 (72.7)	19 (82.6)	
North America	4 (8.9)	1 (4.5)	3 (13.0)	
Asia	6 (13.3)	5 (22.7)	1 (4.3)	
Oceania	0 (0.0)	0 (0.0)	0 (0.0)	

Percentages may not total 100 because of rounding. IQR denotes interquartile range.

**Table 4:** Characteristics of single-site vs. multi-site (**a**) interventional and (**b**) observational trials.

(a)					
	Overall	Single-Center	Multi-Center	N/A	*p*-Value
	(N = 107)	(N = 64)	(N = 36)	(N = 7)	
Trial status, *n* (%)					0.199
Completed	94 (87.9)	58 (90.6)	29 (80.6)	7 (100.0)	
Terminated	13 (12.1)	6 (9.4)	7 (19.4)	0 (0.0)	
Randomized trial, *n* (%)					
No	24 (22.4)	18 (28.1)	4 (11.1)	2 (28.6)	0.136
Yes	83 (77.6)	46 (71.9)	32 (88.9)	5 (71.4)	
Availability of results, *n* (%)					0.890
Yes	84 (78.5)	50 (78.1)	28 (77.8)	6 (85.7)	
No	23 (21.5)	14 (21.9)	8 (22.2)	1 (14.3)	
Published as of 31 December 2023, *n* (%)					0.054
No	36 (33.6)	25 (39.1)	7 (19.4)	4 (57.1)	
Yes	71 (66.4)	39 (60.9)	29 (80.6)	3 (42.9)	
Status and publication, *n* (%)					0.130
Completed and published	62 (57.9)	36 (56.2)	23 (63.9)	3 (42.9)	
Terminated and published	9 (8.4)	3 (4.7)	6 (16.7)	0 (0.0)	
Completed and unpublished	32 (29.9)	22 (34.4)	6 (16.7)	4 (57.1)	
Uncompleted and unpublished	4 (3.7)	3 (4.7)	1 (2.8)	0 (0.0)	
Primary focus, *n* (%)					0.522
Procedures	46 (43.0)	28 (43.8)	15 (41.7)	3 (42.9)	
Devices	26 (24.3)	16 (25.0)	10 (27.8)	0 (0.0)	
Drugs	22 (20.6)	13 (20.3)	7 (19.4)	2 (28.6)	
Diagnostic test	4 (3.7)	2 (3.1)	2 (5.6)	0 (0.0)	
Biomarkers	2 (1.9)	1 (1.6)	1 (2.8)	0 (0.0)	
Other	7 (6.5)	4 (6.2)	1 (2.8)	2 (28.6)	
Median enrollment size [IQR], *n*	200 [60, 801]	133.50 [56.50, 797]	291 [103, 912.50]	90 [37, 537.50]	0.250
Planned sample size ≥100 patients, *n* (%)					0.085
No	38 (35.5)	26 (40.6)	8 (22.2)	4 (57.1)	
Yes	69 (64.5)	38 (59.4)	28 (77.8)	3 (42.9)	
Median follow-up duration [IQR], days	12 [0, 180]	3 [0, 90]	30 [1, 180]	180 [14, 180]	0.165
Follow-up categories, *n* (%)					0.693
0–30 days	85 (79.4)	50 (78.1)	28 (77.8)	7 (100)	
31–365 days	4 (3.7)	3 (4.7)	1 (2.8)	0 (0.0)	
>365 days	18 (16.8)	11 (17.2)	7 (19.4)	0 (0.0)	
Funder type, *n* (%)					0.462
Academic/university	87 (81.3)	52 (81.2)	29 (80.6)	6 (85.7)	
Government	11 (10.3)	9 (14.1)	2 (5.6)	0 (0.0)	
Industry	7 (6.5)	2 (3.1)	4 (11.1)	1 (14.3)	
Non-profit	2 (1.9)	1 (1.6)	1 (2.8)	0 (0.0)	
Median starting date [IQR], year	2015 [2011, 2017.50]	2015.50 [2012, 2018]	2015 [2011, 2017]	2014 [2012, 2015]	0.352
Study period, *n* (%)					0.311
Early	18 (16.8)	11 (17.2)	6 (16.7)	1 (14.3)	
Interim	52 (48.6)	29 (45.3)	17 (47.2)	6 (85.7)	
Late	37 (34.6)	24 (37.5)	13 (36.1)	0 (0.0)	
Median number of sites [IQR], *n*	1 [1, 2.25]	1 [1, 1]	6 [2, 10]	NA [NA, NA]	<0.001
Participating center’s location, *n* (%)					<0.001
Within Europe only	59 (55.1)	38 (59.4)	18 (50.0)	3 (42.9)	
Outside of Europe only	1 (0.9)	0 (0.0)	0 (0.0)	1 (14.3)	
Both within and outside of Europe	6 (5.6)	0 (0.0)	6 (16.7)	0 (0.0)	
N/A	41 (38.3)	26 (40.6)	12 (33.3)	3 (42.9)	
Chairman’s office-primary location (country-detailed), *n* (%)					0.023
France	16 (15.0)	13 (20.3)	3 (8.3)	0 (0.0)	
United States	20 (18.7)	9 (14.1)	11 (30.6)	0 (0.0)	
Korea, Republic of	14 (13.1)	10 (15.6)	1 (2.8)	3 (42.9)	
Sweden	12 (11.2)	6 (9.4)	5 (13.9)	1 (14.3)	
Denmark	8 (7.5)	5 (7.8)	3 (8.3)	0 (0.0)	
Norway	4 (3.7)	3 (4.7)	1 (2.8)	0 (0.0)	
Slovenia	2 (1.9)	2 (3.1)	0 (0.0)	0 (0.0)	
Taiwan	5 (4.7)	5 (7.8)	0 (0.0)	0 (0.0)	
Canada	4 (3.7)	1 (1.6)	3 (8.3)	0 (0.0)	
Finland	2 (1.9)	0 (0.0)	2 (5.6)	0 (0.0)	
Germany	3 (2.8)	2 (3.1)	1 (2.8)	0 (0.0)	
Spain	4 (3.7)	2 (3.1)	1 (2.8)	1 (14.3)	
Netherlands	3 (2.8)	2 (3.1)	1 (2.8)	0 (0.0)	
Italy	1 (0.9)	0 (0.0)	1 (2.8)	0 (0.0)	
United Kingdom	0 (0.0)	0 (0.0)	0 (0.0)	0 (0.0)	
Australia	2 (1.9)	1 (1.6)	1 (2.8)	0 (0.0)	
Belgium	2 (1.9)	0 (0.0)	1 (2.8)	1 (14.3)	
Other	5 (4.7)	3 (4.7)	1 (2.8)	1 (14.3)	
Chairman’s office—primary location (continent), *n* (%)					0.006
Europe	62 (57.9)	38 (59.4)	21 (58.3)	3 (42.9)	
North America	23 (21.5)	10 (15.6)	13 (36.1)	0 (0.0)	
Asia	20 (18.7)	15 (23.4)	1 (2.8)	4 (57.1)	
Oceania	2 (1.9)	1 (1.6)	1 (2.8)	0 (0.0)	
**(b)**					
	**Overall**	**Single-Center**	**Multi-Center**	**N/A**	***p*** **-Value**
	**(N = 45)**	**(N = 29)**	**(N = 10)**	**(N = 6)**	
Trial status, *n* (%)					
Completed	43 (95.6)	29 (100)	8 (80)	6 (100)	0.026
Terminated	2 (4.4)	0 (0.0)	2 (20)	0 (0.0)	
Availability of results, *n* (%)					
Yes	44 (97.8)	28 (96.6)	10 (100.0)	6 (100.0)	0.754
No	1 (2.2)	1 (3.4)	0 (0.0)	0 (0.0)	
Published as of 31 December 2023, *n* (%)					
No	22 (48.9)	14 (48.3)	6 (60.0)	2 (33.3)	0.583
Yes	23 (51.1)	15 (51.7)	4 (40.0)	4 (66.7)	
Status and publication, *n* (%)					0.583
Completed and published	23 (51.1)	15 (51.7)	4 (40.0)	4 (66.7)	
Terminated and published	0 (0.0)	0 (0.0)	0 (0.0)	0 (0.0)	
Completed and unpublished	20 (44.4)	14 (48.3)	4 (40.0)	2 (33.3)	
Uncompleted and unpublished	2 (4.4)	0 (0.0)	2 (20.0)	0 (0.0)	
Primary focus, *n* (%)					0.532
Procedures	17 (37.8)	11 (37.9)	4 (40.0)	2 (33.3)	
Devices	9 (20.0)	5 (17.2)	3 (30.0)	1 (16.7)	
Drugs	0 (0.0)	0 (0.0)	0 (0.0)	0 (0.0)	
Diagnostic test	6 (13.3)	3 (10.3)	2 (20.0)	1 (16.7)	
Biomarkers	7 (15.6)	7 (24.1)	0 (0.0)	0 (0.0)	
Other	6 (13.3)	3 (10.3)	1 (10.0)	2 (33.3)	
Median enrollment size [IQR], *n*	112 [50, 460]	150 [50, 300]	105 [59.50, 290.50]	357 [55.75, 896]	0.846
Planned sample size ≥100 patients, *n* (%)					0.863
No	19 (42.2)	13 (44.8)	4 (40.0)	2 (33.3)	
Yes	26 (57.8)	16 (55.2)	6 (60.0)	4 (66.7)	
Median follow-up duration [IQR], days	5.00 [0.00, 210.00]	7.50 [0.00, 365.00]	3.00 [0.00, 210.00]	7.50 [0.25, 138.50]	0.773
Follow-up categories, *n* (%)					0.513
0–30 days	31 (68.9)	18 (62.1)	7 (70.0)	6 (100.0)	
31–365 days	8 (17.8)	7 (24.1)	1 (10.0)	0 (0.0)	
>365 days	6 (13.3)	4 (13.8)	2 (20.0)	0 (0.0)	
Funder type, *n* (%)					0.476
Academic/university	35 (77.8)	23 (79.3)	7 (70.0)	5 (83.3)	
Government	7 (15.6)	5 (17.2)	1 (10.0)	1 (16.7)	
Industry	3 (6.7)	1 (3.4)	2 (20.0)	0 (0.0)	
Non-profit	0 (0.0)	0 (0.0)	0 (0.0)	0 (0.0)	
Median starting date [IQR], year	2016 [2013, 2018]	2017 [2015, 2018]	2016.50 [2013.25, 2018.75]	2014 [2010.75, 2015]	0.107
Study period, *n* (%)					0.178
Early	5 (11.1)	3 (10.3)	1 (10.0)	1 (16.7)	
Interim	19 (42.2)	10 (34.5)	4 (40.0)	5 (83.3)	
Late	21 (46.7)	16 (55.2)	5 (50.0)	0 (0.0)	
Median number of sites [IQR], *n*	1.00 [1.00, 1.50]	1.00 [1.00, 1.00]	5.50 [2.00, 7.75]	NA [NA, NA]	<0.001
Participating center’s location, *n* (%)					0.527
Within Europe only	33 (73.3)	22 (75.9)	6 (60.0)	5 (83.3)	
Outside of Europe only	11 (24.4)	7 (24.1)	4 (40.0)	0 (0.0)	
Both within and outside of Europe	0 (0.0)	0 (0.0)	0 (0.0)	0 (0.0)	
N/A	1 (2.2)	0 (0.0)	0 (0.0)	1 (16.7)	
Chairman’s office—primary location (country-detailed), *n* (%)					0.275
France	9 (20.0)	5 (17.2)	3 (30.0)	1 (16.7)	
United States	4 (8.9)	2 (6.9)	2 (20.0)	0 (0.0)	
Korea, Republic of	4 (8.9)	3 (10.3)	1 (10)	0 (0.0)	
Sweden	4 (8.9)	2 (6.9)	2 (20.0)	0 (0.0)	
Denmark	3 (6.7)	2 (6.9)	0 (0.0)	1 (16.7)	
Norway	2 (4.4)	2 (6.9)	0 (0.0)	0 (0.0)	
Slovenia	4 (8.9)	4 (13.8)	0 (0.0)	0 (0.0)	
Taiwan	1 (2.2)	1 (3.4)	0 (0.0)	0 (0.0)	
Canada	1 (2.2)	0 (0.0)	1 (10)	0 (0.0)	
Finland	2 (4.4)	1 (3.4)	1 (10)	0 (0.0)	
Germany	1 (2.2)	0 (0.0)	0 (0.0)	1 (16.7)	
Spain	0 (0.0)	0 (0.0)	0 (0.0)	0 (0.0)	
Netherlands	1 (2.2)	1 (3.4)	0 (0.0)	0 (0.0)	
Italy	2 (4.4)	0 (0.0)	0 (0.0)	2 (33.3)	
United Kingdom	3 (6.7)	3 (10.3)	0 (0.0)	0 (0.0)	
Australia	0 (0.0)	0 (0.0)	0 (0.0)	0 (0.0)	
Belgium	0 (0.0)	0 (0.0)	0 (0.0)	0 (0.0)	
Other	4 (8.8)	3 (10.3)	0 (0.0)	1 (16.6)	
Chairman’s office—primary location (continent), *n* (%)					0.502
Europe	35 (77.8)	22 (75.9)	7 (70.0)	6 (100.0)	
North America	4 (8.9)	2 (6.9)	2 (20.0)	0 (0.0)	
Asia	6 (13.3)	5 (17.2)	1 (10.0)	0 (0.0)	
Oceania	0 (0.0)	0 (0.0)	0 (0.0)	0 (0.0)	

Percentages may not total 100 because of rounding. IQR denotes interquartile range.

## Data Availability

Further information is available from the corresponding authors upon reasonable request.
